# Protocol for the Women And Their Children’s Health (WATCH) Study: A Cohort of Pregnancy and Beyond

**DOI:** 10.2188/jea.JE20110079

**Published:** 2012-05-05

**Authors:** Alexis J Hure, Clare E Collins, Warwick B Giles, Ian MR Wright, Roger Smith

**Affiliations:** 1 Mothers and Babies Research Centre, Hunter Medical Research Institute, John Hunter Hospital, New Lambton, New South Wales, Australia; 2 School of Medicine and Public Health, Faculty of Health, The University of Newcastle, Callaghan, New South Wales, Australia; 3 School of Health Sciences, Faculty of Health, The University of Newcastle, Callaghan, New South Wales, Australia; 4 Northern Clinical School, Faculty of Medicine, University of Sydney, Royal North Shore Hospital, St Leonards, New South Wales, Australia; 5 Neonatal Intensive Care Unit, John Hunter Children’s Hospital, New Lambton, New South Wales, Australia

**Keywords:** child, diet, growth, pregnancy, weight

## Abstract

**Background:**

The developmental origins of health and disease is a conceptual framework that helps explain the links between our early life exposures and later health outcomes, and is a burgeoning field of research. In this report, we describe the study protocol used in a prospective cohort of women recruited during pregnancy, with postnatal follow-up of the mothers and offspring.

**Methods:**

The Women And Their Children’s Health (WATCH) cohort (*n* = 180 women) is being conducted at the John Hunter Hospital, Australia (from June 2006). Women attended study visits during pregnancy at 19, 24, 30, and 36 weeks’ gestation. Postnatal follow-up of the women and their offspring occurred at 3-month intervals during the first year after birth and annually thereafter, until age 4 years. Fetal ultrasound scans were performed at each pregnancy visit. Pregnancy and birth data were obtained from hospital records. Data collection has included maternal and child anthropometric, biochemical, dietary, physical activity, socioeconomic, medical, and other variables.

**Conclusions:**

The 2 most novel components of our prospective cohort study are (1) the regular and systematic tracking of fetal and child growth and body composition, starting in the second trimester of pregnancy and continuing to age 4 years, and (2) the detailed maternal and child dietary data collection, including biochemical parameters. Detailed cohorts that collect data on the early nutritional, physiological, and social determinants of health are valuable. Despite its relatively small sample size, many hypotheses on developmental origins can be tested or piloted using data collected from the WATCH cohort.

## INTRODUCTION

The developmental origins of health and disease is a conceptual framework that has been established to help explain the links between our early life exposures and later health outcomes.^1^ Early life exposures span the period from the maternal pre-pregnancy state, through pregnancy, and into childhood. Body size and composition, nutrition, growth, and metabolism have been identified as important predictors of later health and disease.^1^ However, a clearer understanding of the role and mechanisms for each of these predictors is needed.

Retrospective cohorts in the United Kingdom provided the first evidence for associations between birth weight and adult diseases such as coronary heart disease,^2^ type 2 diabetes,^3^ hypertension,^3^^,^^4^ and stroke.^5^ The Barker hypothesis suggested that intrauterine undernutrition, which causes disproportionate fetal growth, results in permanent adjustments to offspring physiology and metabolism, thus predisposing the individual to chronic diseases.^6^ Early work in fetal programming was met with reservations and criticism,^7^ as none of the studies provided an actual measure of nutritional intake in pregnant women or their offspring. Instead, early nutrition was inferred from fetal and infant growth, and fetal growth was itself inferred by the surrogate measurement of birth weight. Thus, even if the findings were accepted as valid, questions were raised as to whether nutrition or some other modifier was the etiologic factor.^7^

The proliferation in experimental, mechanistic, and prospective longitudinal research branching from the original work has helped pave the way for broad acceptance of the developmental origins theory.^1^ Today there are many large birth cohorts, including the Pune Maternal Nutrition Study,^8^ the Southampton Women’s Survey,^9^ Project VIVA,^10^ the Avon Longitudinal Study of Parents and Children (ALSPAC) study,^11^ the Danish National Birth Cohort,^12^ the Norwegian Mother and Child (MoBa) Cohort Study,^13^ and the National Children’s Study (NCS).^14^ These cohorts permit measurement of exposures and confounders prospectively and in more-contemporary populations, where overnutrition and obesity are important considerations. National cohorts collect data on many subjects but cannot perform detailed individual assessments. Hence, there is a niche for smaller cohorts that simultaneously collect data on the early nutritional, physiological, and social determinants of health. Novel hypotheses may be generated and piloted from such datasets.

The primary aim of the Women And Their Children’s Health (WATCH) Study is to test whether maternal nutritional and hormonal factors are important predictors of offspring outcomes such as growth, body composition, and childhood cognition. To date, we have published WATCH data on the micronutrient status of women and their offspring^15^ and have shown that maternal weight gain during pregnancy is an important predictor of fetal body composition.^16^ The aim of this article is to report the study protocol and methods used in this prospective cohort of women during their pregnancy, with follow-up of both mothers and offspring up to 4 years after birth.

## METHODS

### Ethics approval

The WATCH Study received ethics approval from the Hunter New England Human Research Ethics Committee in June 2006. The study’s ethics approval was also registered with the University of Newcastle’s Human Research Ethics Committee. Written informed consent was obtained from all participants (and from fathers) before their enrolment in the study, and written informed consent for on-going study participation was renewed at the 2-year child follow-up. Participants receive pre-paid parking permits and a light meal after fasting blood tests. No other financial incentives are provided for participation.

### Study design

The WATCH Study is a prospective longitudinal cohort spanning pregnancy and early childhood. It is conducted at the John Hunter Public Hospital, a tertiary referral center and major obstetric facility for the Hunter New England region of New South Wales, Australia, with approximately 4000 births per year. The first study visit was scheduled to coincide with the fetal anomaly scan at approximately 18 to 20 weeks’ gestation. The study visits and data collection for the WATCH cohort are summarized in the Table.

**Table.** Data collected at each study visit for the WATCH cohort

Data collected	Pregnancy				Postpartum						
	
	^19wk^ V1	^24wk^ V2	^30wk^ V3	^36wk^ V4	^3mo^ V5	^6mo^ V6	^9mo^ V7	^12mo^ V8	^2yr^ V9	^3yr^ V10	^4yr^ V11
Fetal growth and body composition											
Fetal ultrasound scan	✓	✓	✓	✓							
Anthropometry											
Maternal anthropometry, including current weight, ​ height, skinfold thicknesses, and girths	✓	✓	✓	✓	✓	✓	✓	✓	✓	✓	✓
Maternal pre-pregnancy weight (self-reported)	✓										
Mother’s birth weight (self-reported)	✓										
Child anthropometry, including current weight, length/height, skinfold ​ thicknesses, and girths					✓	✓	✓	✓	✓	✓	✓
Paternal (self-reported) anthropometry, including current weight, ​ height, and waist circumference (optional)					✓						
Biochemistry											
Maternal fasting blood sample	✓			✓	✓	✓			✓	✓	✓
Maternal nonfasting blood sample		✓	✓								
Child nonfasting blood sample (optional)						✓			✓	✓	✓
Blood pressure											
Maternal					✓	✓	✓	✓	✓	✓	✓
Child					✓	✓	✓	✓	✓	✓	✓
Dietary intake											
Maternal nutrient supplementation history	✓										
Maternal 4-day weighed food record (including supplements)	✓			✓	✓	✓			✓		
Maternal food frequency questionnaire	✓			✓	✓	✓			✓		
Maternal nutritional biomarkers	✓			✓	✓	✓			✓	✓	✓
Child 4-day weighed food record						✓			✓		
Infant/child feeding recall questionnaire					✓	✓	✓	✓	✓	✓	
Current child feeding practices questionnaire (previous 24 hours of intake)					✓	✓	✓	✓	✓	✓	
Child 24-hour dietary recall							✓	✓	✓	✓	✓
Child food frequency questionnaire									✓		
Other											
Maternal physical activity questionnaire	✓					✓			✓		
Maternal socioeconomic questionnaire	✓					✓					
Maternal medical history	✓					✓					
Maternal weight-related behaviors questionnaire	✓										
Family lifestyle questionnaire									✓		
Lactation and reproductive history										✓	
Child cognitive and behavioral testing											✓
Child buccal swabs											✓

V1, Visit 1; V2, Visit 2 etc.

### Recruitment and participation

Most participants were recruited by research midwives who approached potential participants in the antenatal clinic of the John Hunter Hospital. Approximately 60% of those approached consented to participate in this study.^17^ In addition, a small number of women were recruited as a result of local media coverage (television, newspaper, radio, and magazine articles) or by word of mouth (friend or family referral). Recruitment spanned the 18-month period between June 2006 and December 2007, and a convenience sample of 180 women was enrolled. Seventy-four per cent of those recruited continued to participate in the study after the 2-year follow-up.

### Participants

All women who were less than 18 weeks’ pregnant were considered eligible to participate, including those receiving antenatal care from private obstetricians or shared care from general practitioners. Participants were required to live within the local or neighboring areas and be able to commute to and from the John Hunter Hospital to attend the scheduled study visits.

### Data collection

#### Fetal growth and body composition

Pregnant women were booked to have 4 fetal ultrasound scans, at approximately 19, 24, 30, and 36 weeks’ gestation (±2 weeks). Ultrasound scans were used to collect data on serial growth and body composition (fat and lean mass) of the fetus in utero. The study ultrasound scans were performed by a team of clinical obstetricians and sonographers. To reduce potential interobserver variation in scan measurements, efforts were made to book each participant with the same obstetrician or sonographer for all 4 ultrasound scans. Confirmation of gestational age and fetal anomaly scanning were undertaken at the first study visit. The date of the last menstrual period was used to calculate the estimated date of delivery (EDD) by applying Naegele’s Rule^18^ unless: (1) menstrual dates were unknown or unreliable, (2) the scan EDD differed by more than 4 days from the menstrual EDD in the first trimester (approximately 6 to 13 weeks), or (3) the scan EDD differed by more than 7 days from the menstrual EDD in the second trimester (13 to 20 weeks). Ultrasound examinations were performed using a GE Voluson (GE Health Care; Australia, New Zealand) ultrasound device with a curvilinear array transducer.

Each ultrasound scan was carried out in accordance with a preset study protocol, which included fetal biometry (biparietal diameter, head circumference, abdominal circumference, and femur length). Biparietal diameter was measured as the standard axial view of the fetal head, including the thalami and third ventricle (including only the calvarial diameter) and excluding the soft tissues of the scalp. Head circumference was measured at the same level as the biparietal diameter and excluded the normal soft tissues of the scalp. Abdominal circumference was obtained from the transverse ultrasound of the fetal abdomen at the level of the fetal stomach and the portal vein and measured the perimeter of the abdomen, including the soft tissues. Measurement of femur length excluded the cartilaginous epiphyses.^19^

Fetal body composition measurements of fat and lean muscle mass were collected on cross-sectional images at the abdomen and mid-thigh. Abdominal fat and lean mass imaging was performed at the level of the standard abdominal circumference measurement. The total area of the fetal abdomen (A1) was calculated, after which the lean abdominal area (A2), which excluded the hyperechoic subcutaneous fat layer, was calculated. The fetal abdominal fat area was calculated as A1 − A2 cm^2^. After measurement of femur length, fetal mid-thigh fat and lean mass were measured over the midpoint of the femur with the transducer rotated 90°.^20^^,^^21^ The total cross-sectional area of the fetal mid-thigh (T1) was calculated, after which the cross-sectional area of the hypoechoic fetal mid-thigh muscle and bone (T2) was calculated. The fetal mid-thigh fat area was calculated as T1 − T2 cm^2^.^21^

#### Anthropometry

With the exception of self-reported maternal pre-pregnancy weight and paternal height, weight, and waist circumference, physical measurements of the mother and children were obtained during the study visits. Maternal and child anthropometric measurements were taken at every study visit by a team of Accredited Practising Dietitians, each with Level One Anthropometrist certification from the International Society for the Advancement of Kinanthropometry (ISAK). Weight, height, length, skinfold thicknesses, and girths were measured, whenever possible, in accordance with the ISAK protocol.^22^ Height and weight were used to calculate body mass index (BMI) using the standard formula: weight (in kg)/height^2^ (in m).

Skinfolds and girths were measured on the right side of the body, irrespective of handedness. Two measurements were taken at each of the specified sites and then averaged. If the second measurement was not within 7.5% of the first measure for skinfold sites, or within 1.5% for girths, a third measure was taken and the median value was used. These precision ranges are slightly higher than the standards set by ISAK, ie, 5% variation for skinfold sites and 1% variation for girths,^22^ due to the inherent difficulties in measuring young children.

#### Weight

Maternal weight was measured using the same set of annually calibrated AND FV-150K electronic weighing scales (A&D Mercury Pty Ltd, Thebarton, South Australia). These scales can weigh individuals up to 150 kg and are accurate to the nearest 50 g. Before being weighed, participants were asked to remove their shoes, excess clothing, and any items from their pockets. Infants 12 months of age or younger were weighed naked at each study visit, using the same set of electronic baby scales (Nuweigh LOG 244, Newcastle Weighing Services, Newcastle, Australia), which are correct to 10 g. Starting at the 2-year child follow-up visit, both mothers and infants were weighed on the same set of annually calibrated NUWEIGH EB8271 electronic weighing scales (NUWEIGH, Wickham, New South Wales), which can weigh individuals up to 150 kg and are accurate to the nearest 100 g.

#### Height

Heights were measured using ISAK procedures for stretch stature. Maternal standing height without shoes was measured to a precision of 1 mm, at 2 study visits, using the same wall-mounted Seca stadiometer (Seca Deutschland, Hamburg, Germany). The 2 readings were averaged, unless the measures differed by more than 1.5%, in which case a third measure was taken and the median value was used. From age 2 years, the standing height of the children without shoes was measured to a precision of 1 mm, in duplicate, using a 200-cm wall-mounted stadiometer.

#### Length

Crown-to-heel length was measured in infants younger than 2 years to a precision of 1 mm using a Harpenden Infant Measuring Table (Holtain Ltd, Crosswell, UK). Each measure was taken in duplicate, and length was calculated as the mean of the 2 measures. If the 2 measures differed by more than 1.5%, length was measured a third time and the median value was used.

#### Skinfolds

**Figure 1.**
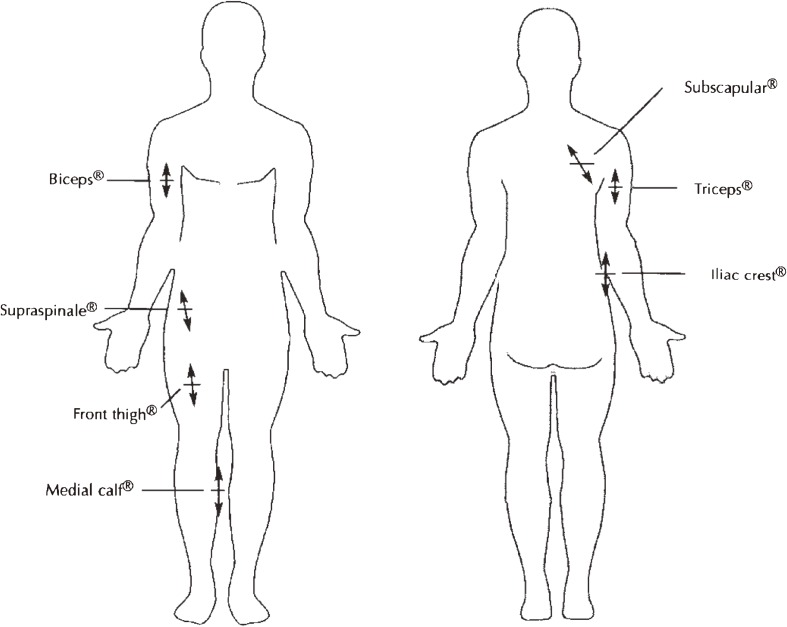
Location of skinfold sites evaluated in the WATCH study, as defined by the International Society for the Advancement of Kinanthropometry. All measurements were made on the right side of the body. Anterior view (left), posterior view (right). Modified from the *International Standards for Anthropometric Assessment*, M Marfell-Jones, T Olds, A Stewart, and L Carter, Anatomical landmarks, p. 27, 2006, with permission from Olds.

Harpenden skinfold calipers (Holtain Ltd, Crosswell, UK) were used to measure all maternal and infant skinfold thicknesses. Maternal skinfold thicknesses were measured at the triceps, subscapular, biceps, supraspinale, front thigh, and medial calf,^22^ as shown in Figure 1. Infant skinfold thicknesses were measured at the subscapular, biceps, iliac crest, and medial calf.^22^

#### Girths

**Figure 2.**
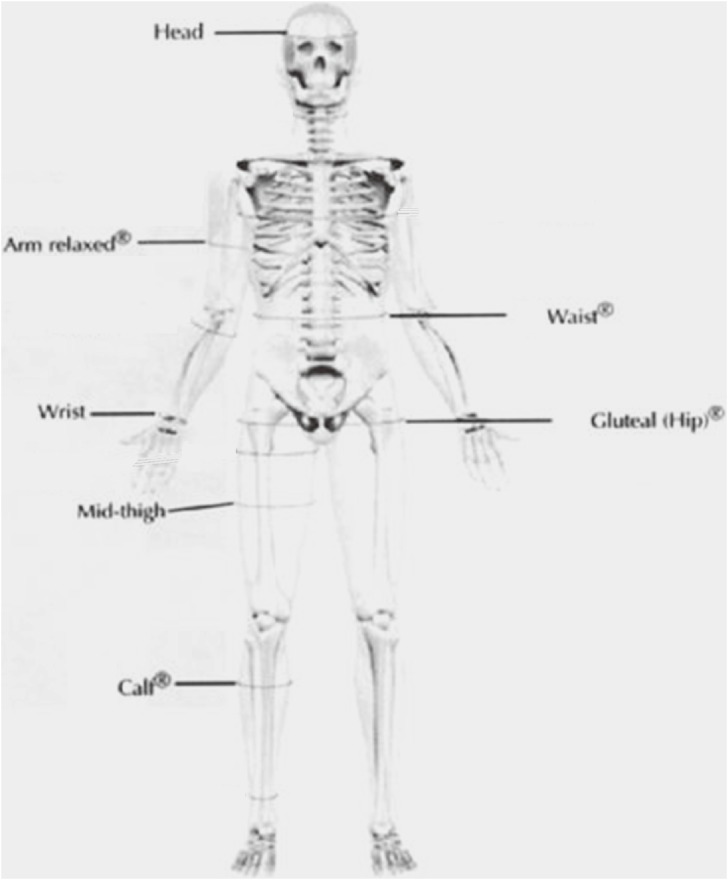
Site of girth measurements, as defined by the International Society for the Advancement of Kinanthropometry. Except for the head, all measurements were made on the right side of the body. Modified from the *International Standards for Anthropometric Assessment*, M Marfell-Jones, T Olds, A Stewart, and L Carter, Girths, p. 78, 2006, with permission from Olds.

Maternal girths were measured at the mid-upper arm (arm relaxed), wrist, waist, gluteal (hip), mid-thigh, and calf^22^ using a Lufkin Executive Thinline flexible steel measuring tape (W606PM, Cooper Hand Tools, NC, USA). This anthropometric tape measures up to 2 m in length and does not stretch with repeated use. The mid-thigh circumference was measured directly in line with the front thigh skinfold site,^22^ at the mid-point between the inguinal fold and the superior border of the patella (while the leg was bent at 90°).^23^ Infant girths were measured at the head, mid-upper arm (arm relaxed), wrist, abdomen (at the level of the umbilicus), mid-thigh, and calf. The locations of the girth measurements are shown in Figure 2.

#### Paternal anthropometry

The mothers were provided with “Father’s Packs” at the 3-month infant follow-up visit, to allow the fathers of the children to provide their current weight, height, and waist circumference. Delivery of the father’s invitation was left to the discretion of the child’s mother. Included in the father’s pack was a WATCH Study information pamphlet, a consent form, a self-report anthropometry recording sheet, and an addressed, reply-paid envelope. Instructions were provided on how to collect and report the data.

#### Biochemistry

Study visit blood samples were collected and assayed by the Hunter Area Pathology Service, which is accredited by the National Association of Testing Authorities. An attempt was also made to have delivery suite staff collect a maternal blood sample during labor and fetal cord blood at birth.

Fasting maternal blood samples were collected after an overnight (approximately 12-hour) fast and have been assayed for total cholesterol, triglycerides, high-density lipoprotein cholesterol (HDL-C), low-density lipoprotein cholesterol (LDL-C; calculated), high-sensitivity C-reactive protein, insulin, glucose, glycosylated hemoglobin (HbA1c), red cell folate, plasma folate, plasma vitamin B12, and plasma homocysteine. Nonfasting maternal blood samples were assayed for hormonal biomarkers. Fetal cord blood samples have had a full blood count performed. At the 6-month infant follow-up, mothers had the option of consenting to the collection of a 5-ml infant nonfasting blood sample, collected by a pediatric phlebotomist. Infant blood samples have undergone a full blood count and been assayed for red cell folate or plasma folate, plasma vitamin B12, and plasma homocysteine. The same child blood tests have been repeated at 2 and 3 years of age. Any remaining plasma or red cells from each collection have been stored.

#### Blood pressure

Systolic blood pressure, diastolic blood pressure, and mean arterial pressure were measured using an automated DINAMAP PRO 300V2 blood pressure monitor (GE Healthcare, Helsinki, Finland) under standardized conditions. Adult subjects were seated for 5 minutes before their blood pressure was measured. Children were seated on their mother’s lap, and a book was read to them. Cuff size was determined from their mid-upper arm girth measurement. Blood pressure was measured once, unless the readings obtained were outside of normal adult or pediatric reference ranges. There was a 2-minute rest period between measurements if a second measurement was required.

#### Dietary intake

A triangulation (or mixed) method of dietary data collection was used to facilitate completeness in dietary representation and to eventually validate the information that is collected in the WATCH Study. Dietary intake was assessed using self-completed food frequency questionnaires (FFQ) and 4-day weighed food records (WFR). Blood samples were collected and assayed for some markers of dietary intake, thereby providing a measure that is independent of human reporting and recording bias.

#### Four-day weighed food records

Bingham et al assessed the validity of weighed food records against other methods of dietary data collection and biological markers and found weighed food records to be the most accurate method of dietary assessment.^24^ Each participant was provided with a set of SOEHNLE Venezia electronic kitchen scales (Soehnle-Waagen GmbH & Co, Murrhardt, Germany) for recording their WFR. The Venezia scales can weigh portions up to 2 kg and are accurate to 1 g. At the first study visit, the study dietitian showed each participant how to use these scales to quantify the amount of each food and beverage item consumed. Food diaries (which included detailed instructions on how to complete the WFR), an example of a 24-hour dietary record, and blank recording sheets were issued with the scales, along with an addressed, reply-paid envelope for return of the records.

Participants were asked to record 4 days of dietary intake within 2 weeks of the study visit. The 4 selected days did not need to be consecutive, and 1 weekend day was to be included. Each day of recorded data was supposed to be representative of current daily intake, and questions relating specifically to vitamin and mineral supplementation and physical activity were included in the data recording sheets.

Weighed food records were analyzed using FoodWorks Professional 2009 (Xyris Software (Australia) Pty Ltd, Brisbane, Queensland), which includes the current nutrient composition tables for use in Australia (AUSTNUT 2007, NUTTAB 2006). The database was supplemented with individualized recipes provided by participants and manufacturers’ information for products not found in the database. Dietary data was entered by an Accredited Practising Dietitian. Energy cut-points were calculated so as to reduce the biases associated with misreporting of dietary intake.

#### Dietary questionnaire for epidemiologic studies

Usual maternal dietary intake was assessed using a FFQ known as the Dietary Questionnaire for Epidemiological Studies (DQES) version 2, or alternatively, the Anti-Cancer Council of Victoria Food Frequency Questionnaire (ACCVFFQ). This questionnaire was developed by the Cancer Council of Victoria, and the items included were those that made important contributions to nutrient intakes in Australian, Greek, and Italian cohort members, as determined by a pilot study.^25^ Both the development of the questionnaire^25^ and its validation in a cohort of young Australian women have been previously reported.^26^

The DQES is a computer-scannable questionnaire purchased at a price that includes translation into a spreadsheet of micro- and macronutrients. The data analysis was carried out by the FFQ distributors, and the results were returned in Microsoft Excel format to facilitate data importation into a statistical software package.

The questionnaire asked respondents to use a 10-point frequency scale to report their usual consumption of 74 foods and 6 alcoholic beverages over the preceding 3 to 12 months. The categories for reporting were: never, less than once per month, once per week, twice per week, 3 to 4 times per week, 5 to 6 times per week, once per day, twice per day, and 3 or more times per day. Questions on total intakes of fruit and vegetables were used to adjust intakes of individual fruits and vegetables, which tend to be overestimated. Portion photographs of vegetables, potatoes, meat, and casserole dishes were used to calculate a portion factor that was applied to scale up or down the standard portions of foods that showed variation by sex or ethnicity in the WFRs from which the FFQ was derived. Additional questions were asked about the number of servings or type of fruit, vegetables, bread, dairy products, eggs, fat spreads, and sugar. Nutrient intakes were computed from the NUTTAB 1995 database, using software developed by the Cancer Council of Victoria.

#### Infant feeding questionnaires

Infant feeding data were recorded at 3-month intervals during the first year and at age 2 years, using a variety of techniques administered by the study’s Accredited Practising Dietitian. The *Infant Feeding Recall* questionnaire collected information on breast feeding initiation and duration (ever, at hospital discharge, and currently). It asked if infant formula, cow’s milk, and other milk substitutes (specified by type) had ever been given regularly, and if semi-solid or solid food had been introduced. The duration of breast and formula feeding, and the age at which these fluids or solids were regularly introduced, were recorded in units of weeks. Questions were selected from the *NSW Child Health Survey 2001*^27^ and the *1995 National Nutrition Survey*^28^ as suggested by Hector and colleagues.^29^

The *Current Feeding Practices* questionnaire recorded the infant’s breast feeding and formula intake and whether the child received any of the following within the previous 24 hours: vitamin or mineral supplements; medicine; plain water; sweetened or flavored water (for example, cordials and soft drinks); fruit juice; tea or infusion; canned, powdered, or fresh milk; solid or semi-solid foods; oral rehydration salts; and other foods or fluids. The *Current Feeding Practices* questionnaire was based on the recommendations of Webb and colleagues.^30^ Following this, a structured 24-hour dietary recall was used to quantify the child’s total dietary intake of beverages and foods from the previous day. Infants were classified as being exclusively, predominantly, complementarily, or not breastfed at each study visit according to the World Health Organization definitions.^31^

#### Australian Child and Adolescent Eating Survey (ACAES)

The ACAES was used to assess the child’s usual dietary intake from age 2 years. The ACAES is a 135-item semi-quantitative FFQ that was previously tested for reliability and relative validity.^32^ It demonstrated acceptable accuracy for ranking nutrient intakes in Australian youths aged 9 to 16 years^32^ and is currently being validated in both adults and younger children. Portion sizes for individual food items were determined using “natural” serving size (eg, a slice of bread) or were derived from the 1995 National Nutrition Survey (unpublished data purchased from the Australian Bureau of Statistics). Women were asked about their child’s frequency of consumption over the previous 6 months. The frequency options ranged from never to 4 or more times per day, but varied depending on the food item.

The AES includes additional questions on the total number of daily servings of fruit, vegetables, bread, dairy products, eggs, fat spreads, sweetened beverages, and snack foods, and asks the type of bread, dairy products, and fat spreads used. Twelve questions related to food-related behaviors, including items on frequency of take-away food consumption and eating while watching television.

#### Physical activity

The Pregnancy Physical Activity Questionnaire (PPAQ) has previously been designed and validated against 7 days of ActiGraph motion sensor data in a small group of pregnant women in the United States.^33^ It is self-administered and takes approximately 10 minutes to complete.^33^ Subjects were asked to report their average time spent participating in 32 activities, classed as household, care-providing, occupational, sports or exercise, transportation, and inactive (sleeping not included) tasks. For each, respondents were asked to select the category that best approximated the amount of time spent, either per day or per week, during the current trimester. Durations ranged from 0 to 6 or more hours per day and from 0 to 3 or more hours per week. There is an open-ended section at the end of the PPAQ that allows respondents to add activities that have not been listed.

The PPAQ was selected because there are very few physical-activity questionnaires that have been developed specifically for women or validated for pregnancy.^33^ The same questionnaire was reissued to participants at the 6-month infant follow-up, with the title changed to Mothers Physical Activity Questionnaire and the timeframe specified as “during the last 3 months”.

#### Psychosocial constructs

The Weight-Related Behaviors Questionnaire, developed in the United States by Kendall et al, was used to assess the following psychosocial constructs: locus of control, self-efficacy, body image, feelings about motherhood, and career orientation.^34^ This was a self-administered questionnaire with Likert scale responses ranging from strongly agree to strongly disagree, from too heavy to too light, and from very satisfied to not at all satisfied. Before analysis, the coding for some items is reversed so that higher scale scores denote higher levels of the construct being measured.^34^

#### Other information

Medical history, medications, and vitamin and mineral supplementation were self-recorded using a single-page questionnaire issued at the first study visit. A similar questionnaire that asked about educational attainment, level of income (self and household), and marital status was also issued. The questions included in both were modeled on some of those found in the Women’s Health Australia surveys.^35^

Birth outcomes, including neonatal anthropometry, were extracted from the Obstetrix database. Additional medical and socioeconomic data recorded in the Obstetrix database were also extracted. Obstetrix is the major repository in New South Wales for recording antenatal information, patient and family history, and birth outcomes.^36^ It was developed by the NSW Department of Health Obstet Consortium, in conjunction with Microsoft and Meridian Health Informatics.^36^

Cognitive testing, performed by a research psychologist, is included at the 4-year child follow-up. The Wechsler Preschool and Primary Scale of Intelligence (WPPSI-III Australian) for ages 4 to 7.3 years is used (PsychCorp, Sydney, Australia). Concurrently, an attending parent (usually the mother) completes the Child Behaviour Checklist for ages 1.5 to 5 years (ACER, Camberwell, Australia).

Buccal (cheek) swabs are collected at the 4-year follow-up and are intended for DNA extraction.

### Statistical analysis

Sociodemographic and birth variables were described according to pregnancy withdrawals and preterm delivery in an effort to address this as a source of bias. Linear regression, including analysis of variance (ANOVA), analysis of covariance (ANCOVA), and linear mixed-models, is the main technique used for data analysis. Linear mixed-models can account for both within-individual and between-individual, or “random”, variation, which results from changes in the experimental condition.^37^ Linear mixed-models are well suited to unbalanced longitudinal datasets, in which some data are missing.^37^ This is common to human studies because of variations in the number of participants attending at each visit, the timing of the study visits, and human error in data collection and recording. Unbalanced datasets are not amenable to an ANOVA.^37^ Post-hoc power calculations are employed before testing each hypothesis, using PS Power and Sample Size (Vanderbilt University, Nashville, TN, USA). The chance of a type I error (or false positive) has been reduced by adjusting for multiple comparisons using Bonferroni correction^15^ or a reduced *P*-value (ie, α = 0.01 instead of 0.05).^16^ Statistical analyses are performed using Intercooled Stata, version 11 (StataCorp LP, College Station, TX, USA) and a 2-sided significance level of 0.05.

### Strengths and limitations

The 2 most novel components of this prospective pregnancy cohort study are (1) the regular and systematic tracking of growth and body composition of mothers and their offspring starting during pregnancy from approximately 19 weeks’ gestation and continuing to 4 years after birth and (2) the collection of detailed maternal and child dietary data, which include biochemical variables. We have attempted to measure a large number of predictor variables, confounding factors, and proxy outcomes for later disease. Care has been taken to use validated tools and techniques, whenever possible, since the commencement of the study. However, our sample size is modest and there are some research questions that will not be adequately powered for analysis. Nevertheless, the WATCH Study has combined multidisciplinary skills from a team with expertise in obstetrics, sonography, endocrinology, nutrition and dietetics, pediatrics, and biochemistry. A large number of hypotheses regarding developmental origins can be tested using the data collected from this cohort, and the data may be pooled with larger international cohorts.
